# Sample Stability and Protein Composition of Saliva: Implications for Its Use as a Diagnostic Fluid

**DOI:** 10.4137/bmi.s607

**Published:** 2008-02-01

**Authors:** Diederik Esser, Gloria Alvarez-Llamas, Marcel P. de Vries, Desiree Weening, Roel J. Vonk, Han Roelofsen

**Affiliations:** Center for Medical Biomics, University Medical Center Groningen, University of Groningen, Groningen, The Netherlands

**Keywords:** saliva, sample stability, biomarkers, proteomics, mass spectrometry, protein breakdown

## Abstract

Saliva is an easy accessible plasma ultra-filtrate. Therefore, saliva can be an attractive alternative to blood for measurement of diagnostic protein markers. Our aim was to determine stability and protein composition of saliva. Protein stability at room temperature was examined by incubating fresh whole saliva with and without inhibitors of proteases and bacterial metabolism followed by Surface Enhanced Laser Desorption/Ionization (SELDI) analyses. Protein composition was determined by sodium dodecyl sulfate polyacrylamide gel electrophoresis (SDS-PAGE) fractionation of saliva proteins followed by digestion of excised bands and identification by liquid chromatography tandem mass spectrometry (LC-MS/MS). Results show that rapid protein degradation occurs within 30 minutes after sample collection. Degradation starts already during collection. Protease inhibitors partly prevented degradation while inhibition of bacterial metabolism did not affect degradation. Three stable degradation products of 2937 Da, 3370 Da and 4132 Da were discovered which can be used as markers to monitor sample quality. Saliva proteome analyses revealed 218 proteins of which 84 can also be found in blood plasma. Based on a comparison with seven other proteomics studies on whole saliva we identified 83 new saliva proteins. We conclude that saliva is a promising diagnostic fluid when precautions are taken towards protein breakdown.

## Introduction

Saliva is a plasma ultra-filtrate that includes specific salivary proteins produced by three major salivary glands (parotid, sub-mandibular and sub-lingual) and other smaller glands (Baum, 1993). Salivary glands produce around 750 ml of fluid each day (Chicharro, 1998). After secretion in the mouth cavity, the fluid is mixed with bacteria, lining cells, nasal secretions and bronchial secretions and is termed whole saliva (Kaufman, 2000; Kaufman, 2002). Whole saliva is easy to collect in a non-invasive way. This makes saliva an attractive alternative to blood testing (Kaufman, 2002; Lawrence, 2002). Compared to blood sampling, whole saliva collection requires no specially trained personnel, can reduce discomfort and anxiety and may simplify serial sample collection. Saliva tests are also safer than blood tests regarding the risk for hepatitis and HIV. As a diagnostic fluid, saliva has been studied in pilot experiments for several pathological conditions, such as celiac disease (Lanander-Lumikari, 2000), rheumatoid arthritis (Helenius, 2005), HIV (Holmstrom, 1990; Malamud, 1992; Matsuda, 1993; Frerichs, 1994), diabetes mellitus (Belazi, 1998; Lopez, 2003), preterm birth (Heine, 2000; Ramsey, 2003), breast cancer (Streckfus, 2005; Streckfus, 2006), sjögren’s syndrome (Ryu, 2006) and for evaluation of hematopoietic stem cell transplantation (Imanguli, 2007). Saliva composition is influenced by several factors, e.g. circadian rhythms, oral health status and exercise (Dawes, 1993; Chicharro, 1998) but also micro organisms and proteases may have a considerable effect on sample stability/protein degradation. Before saliva can be used as a diagnostic fluid for protein markers in the clinic, its stability should be determined. At present there are only three studies on protein stability in saliva samples (Morris, 2002; Ng, 2003; Schipper, 2007). Two of the studies report on the stability of specific proteins i.e. IgA, Lysozyme (Ng, 2003) and IgG (Morris, 2002). One recent study determined overall protein stability of saliva samples stored on ice, at −20 °C and at −80 °C (Schipper, 2007). In the current study we evaluated in detail the overall protein stability of saliva at room temperature over the first four hours after sample collection since this is a critical period where protein breakdown could be expected. The effect of sample handling, inhibition of bacterial growth and inhibition of protease activity on saliva protein stability was examined by comparative profiling with Surface Enhanced Laser Desorption/Ionization Time of Flight Mass Spectrometry (SELDI-TOF-MS) (Merchant, 2000). In addition we studied whole saliva composition. Whole saliva protein composition has been studied using different proteomic strategies (Huang, 2004; Vitorino, 2004; Wilmarth, 2004; Hu, 2005; Xie, 2005; Guo, 2006; Walz, 2006). Xie et al. (Xie, 2005) identified 437 proteins in saliva using free flow electrophoreses. Guo et al. (Guo, 2006) could identify 1381 proteins employing a capillary electrophoresis approach. However, because of the complexity of whole saliva, each proteomics strategy leads to partial overlapping subsets of saliva proteins (Guo, 2006). Therefore, different proteomics strategies contribute to a comprehensive view of the whole saliva proteome. We analyzed whole saliva composition by fractionating saliva proteins on SDS-PAGE followed by LC-MS/MS analyses of digests from cut-out sections of the gel lane. This proteomics approach has not been applied to saliva before. The results of this approach are compared to previous proteomics studies on whole saliva and discussed in terms of protein origin and function.

## Materials and Methods

### Chemicals

Ammonium bicarbonate, triton X-100, azide, phenylmethylsulphonylfluoride (PMSF), EDTA, ditiothreitol (DTT), iodoacetamide, α-cyano-4-hydroxy cinnamic acid diethylamine salt (CHCA), formic acid (FA) and trifluoroacetic acid (TFA) were purchased from Sigma-Aldrich (Steinheim, Germany). Acetonitrile (ACN) and acetone were obtained from Biosolve (Valkenswaard, The Netherlands), leupeptin from Roche (Mannheim, Germany) and ammonium acetate and 2-propanol from Merck (Darmstadt, Germany). MES running buffer and SeeBlue Pre-Stained standard for SDS-PAGE were obtained from Invitrogen (Breda, The Netherlands). Coomassie staining (PageBlue Staining Solution) was from Fermentas (Vilnius, Lithuania). Seq. grade modified trypsin porcine was purchased from Promega (Madison, WI, U.S.A.).

### Saliva collection

Whole saliva was collected from healthy subjects, four male and three female, between 08:00 a.m. and 10:00 a.m. after overnight fasting, to minimize the influence of circadian rhythms and food debris. Subjects were asked to rinse their mouths with water and discard this before sample collection. Saliva was allowed to accumulate in the floor of the mouth. The accumulated saliva was then spit into a polypropylene test tube and this was repeated until enough saliva was collected (Navazesh, 1993). During the collection process the sample tubes were kept on ice.

### Sample pretreatment

Samples were processed according to Hu et al. (Hu, 2005). Briefly, samples were centrifuged for 5 minutes at 1300 g at 4 °C. The pellet was discarded (debris) and the supernatant was centrifuged for 15 minutes at 14000 g at 4 °C. After centrifugation, the supernatant was stored at −20 °C until analysis. Samples were analyzed the same day.

### Sample stability studies

In the first experiment, sample stability was determined in saliva obtained from seven healthy volunteers (four male, three female). Freshly collected saliva samples were either directly processed (time point 0) or left at room temperature for four hrs before processing. Aliquots of the 0 and 4 hrs time points were then analyzed by SELDI-TOF-MS in duplicate (see below).

In a second experiment a unique saliva sample freshly collected from one healthy male volunteer was divided in 3 aliquots of 1.2 ml and incubated for 0, 0.5, 1 and 4 hrs at room temperature with either a) 40 μl of 100 mM sodium azide to inhibit bacterial activity, b) protease inhibitors: 60 μl of 2 mg/ml PMSF in 2-propanol, 1.2 μl of 1 mg/ml leupeptin in water, 12 μl of 100 mM EDTA, or c) no additives (control). Water was added to a final volume of 1.273 ml for all 3 conditions. At each time point an aliquot was taken and treated as described above (sample pre-treatment section). Thereafter, samples were analyzed in triplicate by SELDI-TOF-MS for protein profiling.

CM10 weak cation exchange proteinchip arrays (Ciphergen biosystems, Fremont, CA, U.S.A.) were assembled in a 96 well bioprocessor and the spots were washed two times with 200 μl binding buffer (100 mM NH_4_Ac pH 4.0, 0.05% Triton X-100) for 5 minutes with vigorous shaking. After removing the buffer from the wells, 90 μl binding buffer and 10 μl saliva sample were randomly applied to the spots (in duplicate or triplicate as detailed above). Samples were allowed to incubate for 30 minutes with continuous shaking. Then, they were removed and spots were washed 3 times with 200 μl binding buffer for 5 minutes and once with 200 μl de-ionized water for 5 minutes. The chips were removed from the bioprocessor and air-dried for 15 minutes, followed by two additions (1 μl each) of a 20% solution of CHCA prepared in 50% ACN and 0.5% TFA. Spots were analyzed using the ProteinChip Reader (model PBS II, Ciphergen Biosystems). The mass spectrometer was calibrated using the All-in-One peptide calibration kit (Ciphergen Biosystems) with a focus mass of 6000 Da. Spectra from the saliva samples were collected with the proteinchip software 3.1 (Ciphergen Biosystems) in the mass range 1–20 kDa. Laser intensity was 190, ionsource voltage 20000 V and detector voltage 2150 V. Cluster analysis was performed by Ciphergen Express 3.0 software (Ciphergen Biosystems): a) between samples collected at 0 hr and 4 hr, combining spectra of all seven volunteers measured in duplicate (28 spectra in total) and b) between different time points (0 hrs, 0.5 hrs, 1 hr and 4 hrs) for every condition (control, in presence of azide and in presence of protease inhibitors) measured in triplicate. Before cluster analyses, spectra to be compared were selected, the baseline was subtracted and profiles were normalized using total ion current. Peaks with a signal to noise ratio higher than 5 were selected and were clustered with peaks with similar masses (mass deviation 0.3%) in other profiles with signal to noise ratios higher than 2. The percentage of spectra in which a peak must appear in order to form a cluster was set to 20%. Significant differences (p < 0.05) in peak height of particular masses were calculated.

### Saliva protein composition

A saliva sample freshly obtained from a healthy volunteer was processed immediately after collection as described in the sample pretreatment section. 10 μl of processed saliva were mixed with NuPAGE LDS sample buffer (Invitrogen, Carlsbad, CA, U.S.A.) according to standard protocol from the manufacturer. SDS-PAGE was then performed on a NuPAGE 12% Bis-Tris gel (Invitrogen) run at 200 V for 50 minutes with MES buffer (Invitrogen). Proteins were visualized with Coomassie staining. For protein identification, the whole lane was excised in 30 bands. Each band was cut into small pieces and stored at −20 °C until analysis. Then, they were washed in water and dehydrated in ACN. Reduction was performed by covering gel pieces with 10 mM DTT in 100 mM ammonium bicarbonate for 1 hr at 60 °C. DTT solution was then replaced by 55 mM iodoacetamide in 100 mM ammonium bicarbonate and gel pieces were incubated at room temperature in the dark for 45 minutes. After washing in water and dehydration in ACN, 0.1 μg of trypsin (in 50 mM ammonium bicarbonate) was added and gel pieces were allowed to rehydrate on ice for 20 minutes. Digestion was carried out overnight at 37 °C. Peptides were extracted by treating the gel pieces with 0.1%FA for 30 minutes with continuous shaking. Peptide mixtures were then stored at −20 °C until LC-MS/MS analysis was performed.

Separation of the resulting tryptic peptide mixtures was performed by nano-scale reversed-phase LC-MS/MS. The Agilent 1100 nanoflow/capillary LC system (Agilent, Paolo Alto, CA, U.S.A.) was equipped with a trapping column (5 × 0.3 mm C_18_RP) (Dionex/LC Packings, Amsterdam, The Netherlands) and a nanocolumn (150 × 0.075 mm, C_18_Pepmap) (Dionex/LC Packings). Peptides mixtures were injected into the trapping column at a flow rate of 10 μl/min (3%ACN/0.1%TFA). After 10 minutes the trapping column was switched into the nanoflow system and the trapped peptides were separated using the nanocolumn at a flow rate of 0.3 μl/min in a linear gradient elution from 95%A (3%ACN/0.1%TFA) to 50%B (97%ACN/0.1%TFA) in 70 minutes, followed by an increase up to 80%B in 5 minutes. The eluting peptides were on-line electrosprayed into the QStar XL Hybrid ESI Quadrupole time-of-flight tandem mass spectrometer, ESI-QTOF-MS/MS (Applied Biosystems, Framingham, MA; MDS Sciex, Concord, Ontario, Canada) provided with a nanospray source equipped with a New Objective ESI needle (10 μm tip diameter). Typical values for needle voltage were 2 kV in positive ion mode. The mass spectrometer was set to perform data acquisition in the positive ion mode, typically with a selected mass range of 300–1500 m/z. Peptides with +2 to +4 charge states were selected for tandem mass spectrometry, and the time of summation of MS/MS events was set to be 2 s. The three most abundant charged peptides above a 30 count threshold were selected for MS/MS and dynamically excluded for 60 s with 50 amu mass tolerance.

ProID software (Applied Biosystems) was used to identify proteins from the mass spectrometric datasets according to UniProt database (May 2005, 181,000 entries). Mass tolerance was set to 0.15 Da (MS) and 0.1 Da (MS/MS) and carboxamido-methylation and methionine oxidation were chosen as modifications for database search.

## Results

### Sample stability

**Figure 1:**
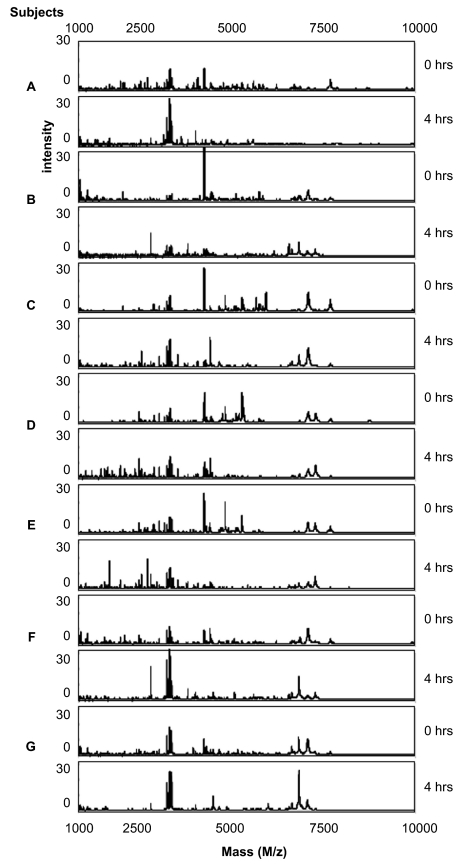
Sample stability at room temperature. Protein profiles of saliva samples of seven volunteers (A–G) taken at 0 and 4 hrs of incubation at room temperature are shown for the mass range of 1000 to 10000 Da. Protein profiles were generated using CM10 proteinchips and 100 mM NH_4_Ac pH 4.0 as binding and washing buffer. CHCA was used as matrix.

**Figure 2:**
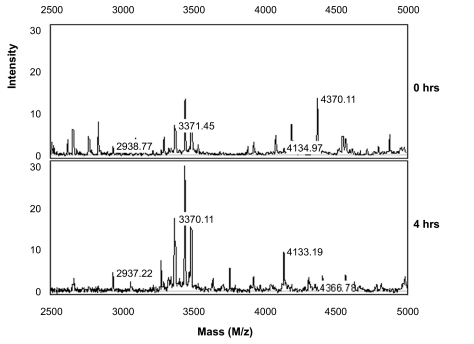
Detailed view of the SELDI profile of volunteer A (see also Fig. 1) in the mass range of 2500 to 5000 Da for saliva samples taken at 0 and 4 hrs of incubation at room temperature. The labeled peaks are discovered degradation markers. The complete list of markers is shown in Table 1.

**Figure 3:**
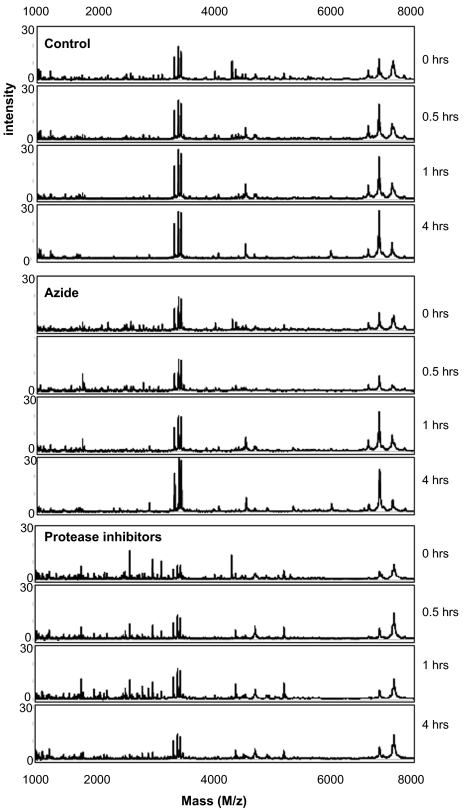
Representative SELDI spectra of a saliva sample incubated at 0, 0.5, 1 and 4 hrs at room temperature in the absence (control) and presence of sodium azide, an inhibitor of bacterial metabolism, and a protease inhibitor cocktail.

**Figure 4:**
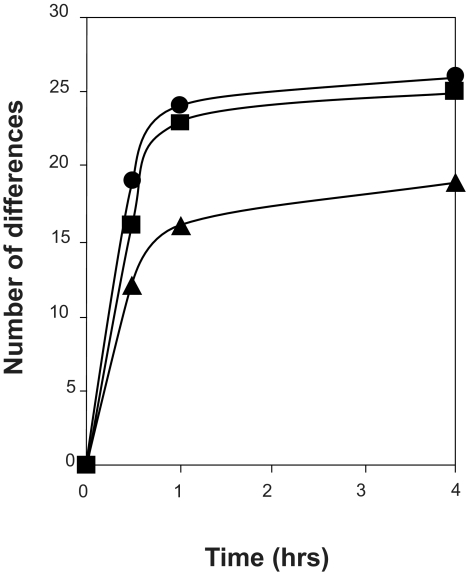
Number of significant differences in peak intensity between the different conditions (• control, + azide and ▴ protease inhibitors) and the different time points compared to 0 hrs. Differences were calculated from the spectra (Fig. 3) for the mass ranges of 1000 to 20000 Da using cluster analyses of triplicate measurements of the samples. Clusters were defined using S/N > 5 (first pass) and S/N > 2 (second pass). Differences were considered significant if p < 0.05.

The stability of saliva at room temperature after sample collection from four male and three female volunteers was evaluated. Freshly collected saliva samples were either directly processed (time point 0) or left at room temperature for 4 hrs and processed as described in the sample pretreatment section. Aliquots taken at the two time points were then spotted in duplicate on CM10 weak cation exchange chips and protein profiles were obtained by SELDI-TOF-MS. Representative spectra obtained from the 1 to 10 kDa range are shown in Figure 1. In this mass range degradation products of larger proteins can be expected. When comparing the protein profiles of fresh samples (0 hrs) of the seven volunteers (A–G) it is evident that there is already considerable variation, especially in the mass range of 1 to 5 kDa between the different individuals. This may be due to biological variation and/or may indicate different degrees of protein degradation between individuals. Also over the period of 4 hrs at room temperature many changes in the spectra can be observed (Fig. 1). To find common peaks that were changed over the 4 hrs period in all seven samples we performed a cluster analyses on the acquired spectra. In total 11 differences were detected and listed in Table 1 together with their fold change in peak intensity between the two conditions. Most peptides are decreased in abundance at 4 hrs, probably because they are further degraded into single amino acids during this period. However, 3 peptides with masses 2937 Da, 3370 Da and 4132 Da are increasing in time which indicates that they are relatively stable breakdown products of larger proteins. Although SELDI technology allows rapid comparison of sample composition, protein/peptide identification is troublesome because it involves purification of each degradation product. Therefore, we attempted to identify the 2937 Da breakdown product by direct SELDI-MS/MS which is possible for peptides with masses below 3000 Da. However, the 2937 Da peptide could not be fragmented by MS/MS even with the highest energy settings and argon as collision gas. This indicates that it is very stable, possibly due to a high degree of post-translational modifications such as glycosylation which may also explain its stability in vivo. As Figure 2 indicates, the three marker peptides can already be detected in “fresh” samples (0 hrs), although with lower intensities. This indicates that breakdown already starts during sample collection and suggests that these markers may be useful indicators of protein breakdown in saliva samples. We examined the protein degradation in saliva in detail to obtain more knowledge on the time frame of the degradation process and whether it was possible to inhibit degradation. Protein degradation could be caused by bacteria in the mouth cavity and/or by proteases present in saliva. Therefore, we studied the influence of sodium azide, an inhibitor of bacterial energy metabolism, and of a protease inhibitor cocktail consisting of PMSF, leupeptin (both serine and cysteine protease inhibitors) and EDTA, an inhibitor of metallo-proteases. Saliva samples were incubated for 0, 0.5, 1 and 4 hrs in the presence and absence of the above mentioned inhibitors. Subsequently, protein profiles were generated (Fig. 3) and compared for differences by cluster analyses as described above. In Figure 4 the number of significant differences is depicted for the different conditions and the different time points compared to 0 hrs (control). For saliva without inhibitors already 19 differences were observed in the first 30 minutes incubation. Thereafter, the number of differences stabilizes. This can be explained by assuming that equilibrium has been reached at this point between the formation of peptides from the breakdown of larger proteins and the degradation of these peptides into single amino acids which are in the mass range of the matrix peaks and therefore are not detected. At 4 hrs 26 differences were observed compared to 0 hrs. This indicates that protein degradation in saliva is a relatively rapid process. It is also clear from Figure 4 that protein breakdown is almost not affected by the addition of azide to the samples, indicating that bacterial metabolism is not contributing much to the protein degradation process, at least for the first hour after collection. The protease inhibitor cocktail is more effective in slowing down the degradation process (Fig. 4). At 4 hrs, 19 differences were observed in the presence of protease inhibitors compared to 26 differences in the control sample. Nevertheless, protein degradation is still substantial with the inhibitor cocktail used. Different inhibitors or combinations of inhibitors need to be evaluated to determine their effectiveness.

### The saliva proteome

**Figure 5:**
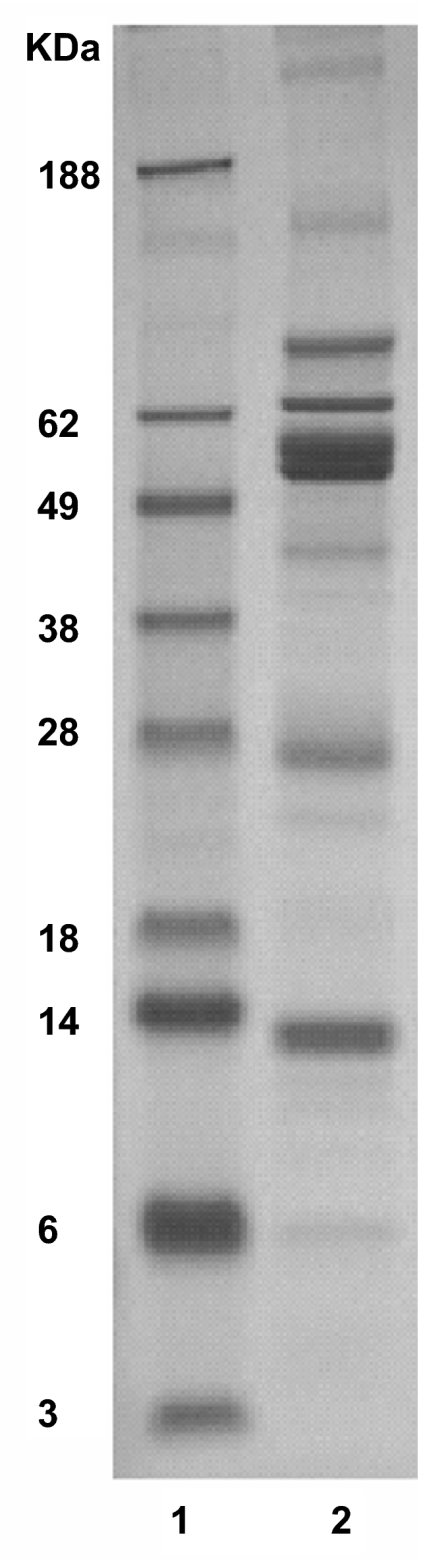
SDS-PAGE of saliva proteins. Lane 1 represents the molecular weight markers. Lane 2 represents proteins present in 10 μl of processed saliva. This lane was cut into 30 bands for further identification by LC-MS/MS.

**Figure 6:**
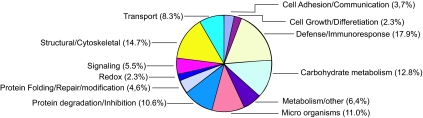
Functional categories of identified proteins, based on information from Swiss Prot, Source and Human Protein Reference Database.

**Figure 7:**
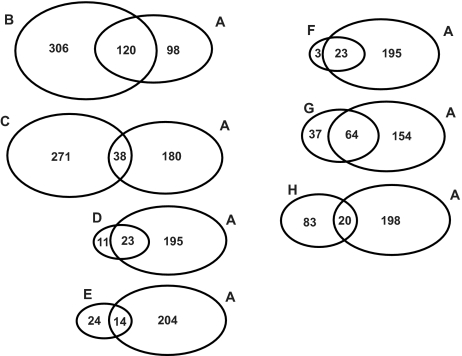
Venn diagrams comparing the proteome results obtained with this study (**A**) versus those achieved by Xie et al. (Xie, 2005) (**B**), Hu et al. (Hu, 2005) (**C**), Vitorino et al. (Vitorino, 2004) (**D**), Walz et al. (Walz, 2006) (**E**), Huang et al. (Huang, 2004) (**F**), Wilmarth et al. (Wilmarth, 2004) (**G**), Guo et al. (Guo, 2006) (**H**). Only part of the data of the study by Guo et al. is publicly available and was used in this comparison.

To determine the saliva composition, saliva proteins were fractionated by SDS-PAGE (Fig. 5). The whole lane was sliced into 30 bands and digested by trypsin. The digests of the bands were subjected to LC-MS/MS for protein identification, as described in detail in the Materials and Methods section. In total we identified 218 proteins, 182 with 99% confidence and 36 with 95% confidence. A complete list of identified proteins is shown in Table 2. Proteins were classified into 12 functional categories based on information from Swiss Prot, Source and Human Protein Reference Database. For each protein also the functional category is listed. In Figure 6 an overview of the different categories is given. The largest category (19.2%) consists of enzymes involved in metabolism, mainly in carbohydrate metabolism (12.8%). This includes enzymes such as α-amylase, lactate dehydrogenase, malate dehydrogenase and fructose-biphosphate aldolase. Another important category (17.9%) includes proteins that are involved in immune response and defense against bacteria. In this group there is a large cluster of IgG chains besides antibactericidal peptides such as dermcidin and bactericidal permeability-increasing protein. Also many proteins from bacterial origin were identified (11% of total). 10.6% of the proteins are involved in degradation. Six proteases were identified in this group e.g. kallikrein, cathepsin D and lysozyme C. Thirteen protease inhibitors are also part of this category such as cystatins, alpha-2-macroglobulin and TIMP-1. The proteases are likely to contribute to the rapid breakdown of saliva proteins that was described above. Also many structural proteins (14.7% of total) were found which are probably derived from cells lining the mouth cavity together with other intracellular proteins that were identified. The transport proteins (8.3% of total) are mainly serum-derived such as albumin, apolipoprotein A-1, transferrin, and ceruloplasmin. Minor categories of proteins are signaling (5.5%), protein modification (4.6%), cell growth and differentiation (2.3%), cell adhesion (3.7%), and proteins involved in maintaining redox status (2.3%). We also compared our results, listed in Table 2, to the HUPO plasma proteome initiative list of 3020 plasma proteins, identified with at least two peptides by LC-MS/MS (www.bioinformatics.med.umich.edu/hupo/ppp). According to the HUPO list, 84 proteins (38.5%) identified in the current study and indicated in Table 2 are also found in plasma. To determine the relevance of the identified proteins, we compared our results to seven other proteomics studies (Huang, 2004; Wilmarth, 2004; Vitorino, 2004; Hu, 2005; Xie, 2005; Walz, 2006; Guo, 2006) on whole saliva composition. Figure 7 shows that there is only partial overlap with our study. Based on this analyses 83 new saliva proteins were identified in our study which are indicated in Table 2.

## Discussion

There is growing interest in using saliva as a diagnostic fluid because of its relatively simple and non-invasive collection procedures. A prerequisite for measuring diagnostic protein markers in saliva is that these proteins are stable in saliva. In this study we show that relatively rapid protein degradation occurs in whole saliva samples at room temperature. We developed a SELDI-based assay to quickly monitor sample integrity. With this assay we show that protein degradation in saliva at room temperature is rapid and starts already during sample collection and handling. Three degradation products with masses of 2937 Da, 3370 Da and 4132 Da were discovered that can be used to monitor the degradation process and to determine the quality of a saliva sample before protein analyses. These markers increase 2 to 7-fold over a period of 4 hrs storage at room temperature, suggesting they are stable breakdown products of larger proteins. The proteome analyses indicates that there are at least six proteases present in saliva (see Table 2) which are probably involved in the observed protein degradation. However, also 13 protease inhibitor proteins were identified which may counteract protease activity. Nevertheless, the overall balance is clearly in favour of degradation. Protein breakdown in saliva could be partly inhibited by a protease inhibitor cocktail targeting serine, cysteine and metallo-proteases. Also in another study, that investigated storage of saliva samples at different temperatures, only partially prevention of degradation was observed with a different inhibitor cocktail (Schipper, 2007). More research is clearly needed to find more effective protease inhibitor cocktails to prevent degradation. A complicating factor in such studies will be that many protease inhibitors are peptides themselves or covalently bind to proteins thereby changing their masses. Both will interfere with proteomics measurements in biomarker discovery studies but may not interfere with ELISA-based measurements of individual proteins. Based on the results of our study we recommend to freeze samples immediately after collection, e.g. in liquid nitrogen, to minimize protein breakdown. Sample processing at 4 °C as well as the use of protease inhibitors can help to reduce degradation. Based on a study by Schipper et al. (Schipper, 2007) long time storage at −80 °C is recommended.

Several different strategies have been employed to analyze the saliva proteome such as 2 D gel (Huang, 2004; Wilmarth, 2004; Vitorino, 2004; Hu, 2005; Walz, 2006), capillary iso-electric focusing (Guo, 2006), and free flow electrophoresis (Xie, 2005). Our approach was to fractionate saliva proteins by SDS-PAGE followed by LC-MS/MS for protein identification. Overall 218 proteins were identified by this proteomics strategy, not applied to saliva before. From the identified proteins we deduced the main functions. These are: carbohydrate-breakdown, immune response/defence against bacteria, and protein degradation. By comparing our results with seven previous proteomics studies (Fig. 7) on whole saliva composition we find only partial overlap with our study. 83 new saliva proteins from our study which were not previously identified are indicated in Table 2. These results show that with each proteomics strategy, partial overlapping subsets of saliva proteins are identified. Therefore, different proteomic approaches will contribute to a more comprehensive view of the saliva proteome. Many of the identified proteins are also found in plasma. Comparison with the HUPO plasma proteome database learned that 38.5% of the identified proteins can also be found in plasma. This relatively high percentage of plasma proteins in saliva illustrate the possibilities for use of saliva as an alternative to blood for diagnosis and biomarker discovery. However, protein breakdown in saliva samples poses a serious problem for quantitative measurements. We conclude that saliva can be a promising diagnostic fluid when precautions are taken towards protein breakdown.

## Figures and Tables

**Table 1:** Masses with significantly different peak intensities between 0 and 4 hrs of incubation of whole saliva at room temperature.

Peak (m/z)	p-value	Fold change
2937	0.0017	6.8
3370	0.025	2.0
4132	0.047	3.0
4368	0.0017	−6.1
4928	0.0017	−17.5
5210	0.018	−3.7
5376	0.0017	−16.5
5839	0.018	−2.8
7751	0.0017	−6.1
10422	0.0088	−5.0
15495	0.0017	−4.9

Clusters were determined using S/N > 5 (first pass) and S/N > 2 (second pass). Differences were considered significant if p < 0.05.

**Table 2:** List of proteins identified with 99% and 95% confidence in human saliva.

Protein name	Accession nr	Function	Mass (Da)
**99% confidence:**			
14-3-3 protein beta/alpha	P31946	Signalling	27951
14-3-3 protein zeta/delta§	P63104	Signalling	27745
6-phosphogluconate dehydrogenase, decarboxylating	P52209	Energy/metabolism	53009
78 kDa glucose-regulated protein precursor§	P11021	Protein Folding/Repair	72333
Actin§	P60709	Structural/Cytoskeletal	41737
Actin-like protein 3#	P61158	Structural/Cytoskeletal	47240
Actin-related protein 2/3 complex subunit 4#	P59998	Structural/Cytoskeletal	19536
Adenine phosphoribosyltransferase	P07741	Energy/metabolism	19477
Adenosylhomocysteinase#	P23526	Energy/metabolism	47585
Alcohol dehydrogenase [NADP+]#	P14550	Energy/metabolism	36442
Alcohol dehydrogenase class IV mu/sigma chain#	P40394	Energy/metabolism	40006
Aldehyde dehydrogenase, dimeric NADP-preferring#	P30838	Energy/metabolism	50379
Aldo-keto reductase family1 member B10	O60218	Energy/metabolism	36021
Alpha enolase§	P06733	Energy/metabolism	47038
Alpha-1-acid glycoprotein 1 precursor§,#	P02763	Defense/Immunoresponse	23512
Alpha-1-antitrypsin precursor§	P01009	Protein Degradation/Inhibitor	46737
Alpha-actinin 1§	P12814	Structural/Cytoskeletal	103058
Apolipoprotein A-I precursor§	P02647	Transport	30778
Arginase 1§	P05089	Energy/metabolism	34735
ATPase 4, plasma membrane-type#	Q9SU58	Micro organism	105718
Bactericidal permeability-increasing protein precursor#	P17213	Transport	53396
Calgranulin B§	P06702	Cell Adhesion/Communication	13242
Carbonic anhydrase VI precursor§	P23280	Energy/metabolism	35365
Carboxylesterase 2 precursor	O00748	Energy/metabolism	61807
Cathepsin D precursor	P07339	Protein Degradation/Inhibitor	44552
Ceruloplasmin precursor§	P00450	Transport	122205
Chaperone protein dnaK#	Q7NXI3	Micro organism	69122
Chitinase 3-like protein 2 precursor#	Q15782	Cell Growth/Differentiation	43501
Chloride intracellular channel protein 1	O00299	Transport	26792
Clusterin precursor§	P10909	Cell Growth/Differentiation	52495
Cofilin, non-muscle isoform	P23528	Structural/Cytoskeletal	18371
Complement C3 precursor§	P01024	Signalling	187164
Complement C4 precursor§	P01028	Defense/Immunoresponse	192771
Complement factor H precursor§,#	P08603	Energy/metabolism	139070
Coronin-1A	P31146	Structural/Cytoskeletal	50895
Cystatin A§	P01040	Protein Degradation/Inhibitor	11006
Cystatin B§	P04080	Protein Degradation/Inhibitor	11140
Cystatin C precursor§	P01034	Protein Degradation/Inhibitor	15799
Cystatin D precursor	P28325	Protein Degradation/Inhibitor	16080
Cystatin S precursor	P01036	Protein Degradation/Inhibitor	16214
Cystatin SA precursor	P09228	Protein Degradation/Inhibitor	16445
Cystatin SN precursor	P01037	Protein Degradation/Inhibitor	16362
Dermcidin precursor§,#	P81605	Defense/Immunoresponse	11284
Desmocollin-2 precursor	Q02487	Cell Adhesion/Communication	99962
Desmoglein-3 precursor	P32926	Cell Adhesion/Communication	107503
Diablo homolog, mitochondrial precursor#	Q9NR28	Signalling	27131
Dihydroxy-acid dehydratase#	Q8XWR1	Micro organism	58965
Dipeptidyl peptidase IV§,#	P27487	Protein Degradation/Inhibitor	88279
DNA polymerase IV#	Q9JYS8	Micro organism	35966
Elongation factor 1-alpha§	P68104	Protein Synthesis	50141
Elongation factor 1-gamma	P26641	Protein Synthesis	49988
Ezrin§,#	P15311	Cell Growth/Differentiation	69268
F-actin capping protein beta subunit	P47756	Structural/Cytoskeletal	31219
Farnesyl pyrophosphate synthetase#	P14324	Energy/metabolism	40532
Fatty acid-binding protein, epidermal	Q01469	Energy/metabolism	15033
Fibrinogen gamma chain precursor§	P02679	Protein Modification/Polymerization	51512
FixC protein#	Q8Z9K9	Micro organism	45687
Fructose-bisphosphate aldolase A§	P04075	Energy/metabolism	39289
Fructose-bisphosphate aldolase C	P09972	Energy/metabolism	39325
Galectin-3 binding protein precursor§	Q08380	Cell Adhesion/Communication	65331
Galectin-7§	P47929	Cell Adhesion/Communication	14944
Gelsolin precursor§	P06396	Structural/Cytoskeletal	85698
Genome polyprotein#	P17593	Micro organism	255497
Glucose-6-phosphate isomerase§	P06744	Energy/metabolism	63016
Glutaminyl-tRNA synthetase#	Q8EG26	Micro organism	64103
Glutathione S-transferase P	P09211	Signalling	23225
Glyceraldehyde-3-phosphate dehydrogenase 1	P04406	Energy/metabolism	35922
Haptoglobin precursor	P00738	Transport	45205
Heat shock 70 kDa protein 1§	P08107	Protein Folding/Repair	70052
Heat shock cognate 71 kDa protein§	P11142	Protein Folding/Repair	70898
Hemoglobin alpha chain	P69905	Transport	15126
Hemoglobin beta chain	Q9UK54	Transport	13964
Hemopexin precursor§	P02790	Transport	51676
Hurpin	Q9UIV8	Protein Degradation/Inhibitor	44276
Hypothetical 84.6 kDa protein#	Q04263	Micro organism	84602
Ig alpha-1 chain C region	P01876	Defense/Immunoresponse	37655
Ig alpha-2 chain C region§	P01877	Defense/Immunoresponse	36508
Ig gamma-1 chain C region§	P01857	Defense/Immunoresponse	36106
Ig gamma-2 chain C region§	P01859	Defense/Immunoresponse	35885
Ig heavy chain V region MOPC 47A#	P01786	Defense/Immunoresponse	12975
Ig heavy chain V-II region NEWM#	P01825	Defense/Immunoresponse	12790
Ig heavy chain V-III region GAL§,#	P01781	Defense/Immunoresponse	12731
Ig heavy chain V-III region HIL#	P01771	Defense/Immunoresponse	13566
Ig heavy chain V-III region TUR§,#	P01779	Defense/Immunoresponse	12431
Ig heavy chain V-III region VH26 precursor#	P01764	Defense/Immunoresponse	12582
Ig kappa chain C region#	P01834	Defense/Immunoresponse	11609
Ig kappa chain V-I region CAR§	P01596	Defense/Immunoresponse	11608
Ig kappa chain V-I region WEA§,#	P01610	Defense/Immunoresponse	11704
Ig kappa chain V-I region	P01611	Defense/Immunoresponse	11840
Ig kappa chain V-III region B6§,#	P01619	Defense/Immunoresponse	11636
Ig kappa chain V-III region GOL§	P04206	Defense/Immunoresponse	11830
Ig kappa chain V-IV region Len§	P01625	Defense/Immunoresponse	12640
Ig lambda chain C regions#	P01842	Defense/Immunoresponse	11237
Ig lambda chain V-I region NEW#	P01701	Defense/Immunoresponse	11453
Ig lambda chain V-I region WAH§,#	P04208	Defense/Immunoresponse	11725
Ig lambda chain V-III region LOI§	P80748	Defense/Immunoresponse	11935
Ig lambda chain V-III region SH§	P01714	Defense/Immunoresponse	11393
Ig lambda chain V-IV region Hil	P01717	Defense/Immunoresponse	11517
Ig mu chain C region	P01871	Defense/Immunoresponse	49557
Immunoglobulin J chain§	P01591	Defense/Immunoresponse	15594
Interleukin-1 receptor antagonist prec.	P18510	Defense/Immunoresponse	20055
Kallikrein 1 precursor	P06870	Protein Degradation/Inhibitor	28890
Keratin, type I cuticular HA3-II§,#	Q14525	Structural/Cytoskeletal	46214
Keratin, type I cytoskeletal 10	P13645	Structural/Cytoskeletal	59519
Keratin, type I cytoskeletal 14§,#	P02533	Structural/Cytoskeletal	51490
Keratin, type I cytoskeletal 16§	P08779	Structural/Cytoskeletal	51137
Keratin, type I cytoskeletal 9§	P35527	Structural/Cytoskeletal	62129
Keratin, type I microfibrillar 48 kDa#	P02534	Structural/Cytoskeletal	46674
Keratin, type II cuticular HB4#	Q9NSB2	Structural/Cytoskeletal	64895
Keratin, type II cytoskeletal 1#	P04104	Structural/Cytoskeletal	65092
Keratin, type II cytoskeletal 1§	P04264	Structural/Cytoskeletal	65886
Keratin, type II cytoskeletal 2 epidermal§	P35908	Structural/Cytoskeletal	65865
Keratin, type II cytoskeletal 4§	P19013	Structural/Cytoskeletal	57285
Keratin, type II cytoskeletal 5§	P13647	Structural/Cytoskeletal	62447
Keratin, type II cytoskeletal 6A	P02538	Structural/Cytoskeletal	59914
Keratin, type II cytoskeletal 6D#	P48667	Structural/Cytoskeletal	42468
Keratin, type II cytoskeletal 6E	P48668	Structural/Cytoskeletal	59894
Keratin, type II microfibrillar, component 7C#	P15241	Structural/Cytoskeletal	53682
Lactoperoxidase precursor	P22079	Redox	80288
Lactotransferrin precursor§	P02788	Transport	78182
Leukotriene A-4 hydrolase	P09960	Energy/metabolism	69154
L-lactate dehydrogenase A chain§	P00338	Energy/metabolism	36558
L-lactate dehydrogenase B chain§	P07195	Energy/metabolism	36507
Long palate, lung and nasal epith. carc.ass. prot.1prec.	Q8TDL5	Defense/Immunoresponse	52442
L-plastin§	P13796	Structural/Cytoskeletal	70158
Lysozyme C precursor	P61626	Protein Degradation/Inhibitor	16537
Macrophage capping protein	P40121	Structural/Cytoskeletal	38518
Malate dehydrogenase, cytoplasmic	P40925	Energy/metabolism	36295
Maspin precursor	P36952	Protein Degradation/Inhibitor	42138
Matrix metalloproteinase-9 precursor§	P14780	Protein Degradation/Inhibitor	78427
Maturase K#	Q9GI85	Micro organism	61017
Metalloproteinase inhibitor 1 prec.§	P01033	Protein Degradation/Inhibitor	23171
Moesin	P26038	Structural/Cytoskeletal	67689
Monocyte differentiation antigen CD14 precursor§,#	P08571	Defense/Immunoresponse	40076
Mucin 5B precursor	Q9HC84	Cell Adhesion/Communication	590499
Myeloperoxidase precursor	P05164	Defense/Immunoresponse	83869
Myoglobin#	P02144	Transport	17053
Myosin heavy chain, non-muscle type A§	P35579	Structural/Cytoskeletal	226401
N-acetylglucosamine kinase#	Q9UJ70	Energy/metabolism	37244
Neutrophil gelatinase-associated lipocalin prec.§	P80188	Transport	22588
Outer membrane usher protein pefC precursor#	P37868	Micro organism	86370
Peptidyl-prolyl cis-trans isomerase A	P62937	Protein Folding/Repair	17881
Peroxiredoxin 5, mitochondrial precursor	P30044	Redox	22026
Peroxiredoxin 6	P30041	Redox	24904
Phosphatidylethanolamine-binding protein	P30086	Protein Degradation/Inhibitor	20926
Phosphoglucomutase§,#	P36871	Energy/metabolism	61318
Phosphoglycerate kinase 1	P00558	Energy/metabolism	44483
Phosphoglycerate mutase 1	P18669	Energy/metabolism	28673
Phospholipid transfer protein prec.§,#	P55058	Energy/metabolism	54739
Plasminogen activator inhibitor-2 prec.#	P05120	Signalling	46596
Polymeric-immunoglobulin receptor precursor	P01833	Defense/Immunoresponse	83314
Proactivator polypeptide precursor	P07602	Protein Degradation/Inhibitor	58113
Profilin-1§	P07737	Structural/Cytoskeletal	14923
Prolactin-inducible protein precursor§	P12273	Defense/Immunoresponse	16572
Proline-rich protein 3 precursor peptide P-B)	P02814	Unknown	8188
Prominin 1 precursor#	O43490	Signalling	97202
Protein-glutamine glutamyltransferase E prec.§	Q08188	Energy/metabolism	76632
Purine nucleoside phosphorylase	P00491	Energy/metabolism	32118
Pyruvate kinase, isozymes M1/M2§	P14618	Energy/metabolism	57806
Rab GDP dissociation inhibitor beta	P50395	Signalling	50663
Ras-related C3 botulinum toxin substrate 2#	P15153	Transport	21429
Rho GDP-dissociation inhibitor 2	P52566	Signalling	22857
S100 calcium-binding protein A7§	P31151	Cell Growth/Differentiation	11326
Salivary alpha-amylase precursor	P04745	Energy/metabolism	57768
Serine/threonine-protein kinase BRI1- like 2 precursor#	Q9ZPS9	Energy/metabolism	39280
Serine/threonine-protein kinase RIPK4#	P57078	Energy/metabolism	91611
Serotransferrin precursor§	P02787	Transport	77050
Short palate, lung and nasal epith.carc.ass.prot.2prec.	Q96DR5	Transport	27011
Small proline-rich protein 3	Q9UBC9	Structural/Cytoskeletal	18154
SPARC-like protein 1 precursor	Q14515	Protein Degradation/Inhibitor	75216
Squamous cell carcinoma antigen 1§	P29508	Protein Degradation/Inhibitor	44565
Sugar fermentation stimulation protein homolog#	Q97VP5	Micro organism	27830
Thioredoxin§	P10599	Redox	11606
Transaldolase	P37837	Energy/metabolism	37540
Transcobalamin I precursor	P20061	Transport	48195
Transgelin-2	P37802	Structural/Cytoskeletal	22260
Transketolase§	P29401	Energy/metabolism	67878
Triosephosphate isomerase isomerise§	P60174	Protein Folding/Repair	26538
Tyrosine recombinase xerC	Q8UC70	Micro organism	34532
Ubiquitin#	O46543	Protein Degradation/Inhibitor	8583
Von Ebner’s gland protein precursor	P31025	Transport	19250
Zinc-alpha-2-glycoprotein precursor§	P25311	Energy/metabolism	33872
**95% confidence proteins**			
14-3-3 protein sigma§	P31947	Signalling	27774
30S ribosomal protein S20#	Q7VQL2	Micro organism	10264
50S ribosomal protein L5#	Q8CRH2	Micro organism	20236
Acetyl-CoA acetyltransferase#	P45359	Energy/metabolism	41241
Alanyl-tRNA synthetase#	Q971J4	Micro organism	103675
Alpha-2-macroglobulin precursor§	P01023	Protein Degradation/Inhibitor	163278
Bact.l/permeability-increasing protein- like 1 prec.	Q8N4F0	Transport	49172
Beta crystallin B1 (Beta-35)#	P53674	Cell Growth/Differentiation	27892
Carbonyl reductase [NADPH] 1#	P16152	Energy/metabolism	30244
Carcinoembryonic antigen-related cell adh. mol.5prec.§,#	P06731	Defense/Immunoresponse	76796
Catalase§	P04040	Redox	59625
CD9 antigen (p24)#	P21926	Cell Adhesion/Communication	25285
Chaperone protein htpG	P61185	Micro organism	73731
Cystatin B#	Q862Z5	Protein Degradation/Inhibitor	11103
Dihydrolipoyllysine-residue succinyltransferase#	P36957	Energy/metabolism	48640
Ethanolamine utilization protein#	P41793	Micro organism	49174
F-actin capping protein alpha-2 subunit#	P47755	Structural/Cytoskeletal	32818
Ferredoxin II#	P00237	Micro organism	9962
Genome polyprotein#	P17594	Micro organism	255428
Glucose-6-phosphate 1-dehydrogenase#	P11413	Energy/metabolism	59135
Heat shock protein HSP 90-beta#	P08238	Protein Folding/Repair	83133
Hut operon positive regulatory protein#	P10943	Micro organism	16064
Hypothetical protein ynaA#	P77658	Micro organism	37060
Hypothetical UPF0135 protein CPn0137#	Q9Z946	Micro organism	27236
Ig heavy chain V region UPC10§,#	P01811	Defense/Immunoresponse	13001
Ig kappa chain V-II region TEW§	P01617	Defense/Immunoresponse	12316
Ig kappa chain V-III region NG9 precursor§,#	P01621	Defense/Immunoresponse	10729
Myeloblastin precursor	P24158	Protein Degradation/Inhibitor	27807
Potential phospholipid-transporting ATPase VA#	O60312	Energy/metabolism	167688
Probable Na(+)/H(+) antiporter nhx-9	P35449	Micro organism	75281
Probable serine/threonine-protein kinase#	P28966	Micro organism	65248
Pyruvate kinase, isozymes R/L#	P30613	Energy/metabolism	61830
Rho GDP-dissociation inhibitor 1	P52565	Signalling	23076
Serum albumin precursor§	P02768	Transport	69367
Vinculin#	P18206	Cell adhesion/Communication	123668
Zinc finger protein 446#	Q9NWS9	Signalling	48957

§ Proteins that are also found in plasma according to the HUPO Plasma Proteome Initiative list of plasma proteins (www.bioinformatics.med.umich.edu/app/hupo/ppp/).

# Novel saliva proteins identified in this study compared to seven previous studies (see Fig. 7).
